# The role of psychological autopsy in investigating a case of atypical suicide in schizophrenia: a case report with a brief review of literature

**DOI:** 10.1186/s41935-022-00291-5

**Published:** 2022-07-06

**Authors:** Roxana-Mihaela Crișan, Ciprian Ionuț Băcilă, Silviu Morar

**Affiliations:** 1 Doctoral Department, Faculty of Medicine, Lucian Blaga University From Sibiu, 550024 Sibiu, Romania; 2 Forensic Department, County Clinical Emergency Hospital of Sibiu, Corneliu Coposu Boulevard 2–4, 550245 Sibiu, Romania; 3 Dental Medicine and Nursing Department, Faculty of Medicine, Lucian Blaga University From Sibiu, “Gh. Preda” Clinical Psychiatry Hospital, 550024 Sibiu, Romania; 4 Preclinical Department, Faculty of Medicine, Lucian Blaga University From Sibiu, 550024 Sibiu, Romania

**Keywords:** Forensic autopsy, Atypical suicide, Psychological autopsy, Schizophrenia, Risk assessment, COVID-19 pandemic

## Abstract

**Background:**

Self-harm typically is without lethal intent. Death can occur rarely, with suicide taking on an atypical form that raises the suspicion of hetero-aggression. Our study aimed to identify the link between self-harm and suicide intent and also to outline the positive diagnosis of an atypical suicide case which has raised the suspicion of hetero-aggression. For this purpose, the psychological autopsy method should be used regularly in suicide investigation because it not only allows a positive diagnosis of suicide but can also provide a detailed picture of mental degradation and associated suicide risk factors.

**Case presentation:**

The case of a 26-year-old man from a rural area, found dead in the basement, at home, naked, barricaded inside, is described.

**Methods:**

The on-site investigation and a complete forensic autopsy were performed. In addition, we apply the psychological autopsy method which gathered enough information to outline the positive diagnosis of suicide. We also made a brief literature review on the suicide risk factors and the behavioral changes that occurred during the COVID-19 pandemic in schizophrenic patients.

**Results:**

The forensic autopsy revealed that he presented a complex craniofacial trauma as the cause of death (with scalp lacerations, frontal fracture, subarachnoid hemorrhage, and frontal cerebral contusions) associated with torso trauma (with self-inflicted stabbed wounds) with bruises and abrasions on the limbs. The injuries that caused death were self-inflicted and ensued repeatedly hitting his head against blunt objects. Using the psychological autopsy method, we found out that he presented multiple psychiatric hospitalizations for schizophrenia for almost 10 years, recently with reduced compliance to treatment. We also documented two previous suicide attempts and a gradual deterioration of his mental health.

**Conclusions:**

We highlighted the role of the psychological autopsy (in addition to the judicial investigation and the forensic autopsy) for the diagnosis of committed suicide, for making a rigorous differential diagnosis between accident, hetero-aggression, and suicide, and also in pin-pointing the suicide risk factors.

**Supplementary Information:**

The online version contains supplementary material available at 10.1186/s41935-022-00291-5.

## Background

Suicide is a serious public health problem. There is a link between mental disorders and committed suicide (World Health Organization 2021). Most people can control their suicidal ideation and do not commit suicide. The practice guidelines of the Psychiatric Association for the Psychiatric Assessment of Adults state that suicidal ideation is a symptom of a psychiatric disorder, and 90% of those who had completed suicide meet the criteria for a psychiatric condition (Harmer et al. 2021).

The suicide rate in people with schizophrenia is about 10% (Sher and Kahn 2019). The risk of suicide is difficult to assess in these cases. Depression, as well as suicide attempts, are the main risk factors. Young men seem to be more vulnerable during inpatient care (Sinclair et al. 2004). In a study on 288 schizophrenic patients, almost half (49%) reported lifelong self-harm during the interview. This study also revealed that self-harm is widespread in both genders (Mork et al. 2012). The intention in self-harm is not suicide, it can coexist with suicidal ideation, and it can be a strong predictor of completed suicide (Mork et al. 2012). It is common knowledge that people with a history of self-harm are at a higher risk of suicide. The relationship between self-harm and suicide proved to be a complex one (Chan et al. 2016).

The use of psychological autopsy might be of paramount importance in identifying people at risk of suicide, outlining a socio-psychological or psychopathological profile. This method allows the detection and understanding of the circumstances preceding the suicide: (1) suicidal motives, (2) sociological and family parameters, and (3) self-destructive behavior (Hădărean et al. 2011; Majid et al. 2017).

During a pandemic, people with mental health disorders are more vulnerable, with a greater need for appropriate mental health services (Neelam et al. 2021). The COVID-19 pandemic has negative consequences on their mental health. At the same time, the COVID-19 pandemic has created a challenge for healthcare professionals in managing patients with schizophrenia. Treatment continuity is crucial to prevent mental and physical deterioration in these patients (Kozloff 2020).

In cases of committed suicide, the forensic approach provides a systematic analysis of all objective elements. Consequently, the gathered data play an essential role in the differential diagnosis of suicide versus homicide or accidental death. This case emphasizes the importance of the psychological autopsy in confirming the positive diagnosis of suicide by investigating the psychiatric past. Furthermore, during the case discussion, we reviewed the literature on suicide risk factors and the behavioral changes during the COVID-19 pandemic in schizophrenic patients.

## Case presentation

### Circumstances at the place of death

**Fig. 1:**
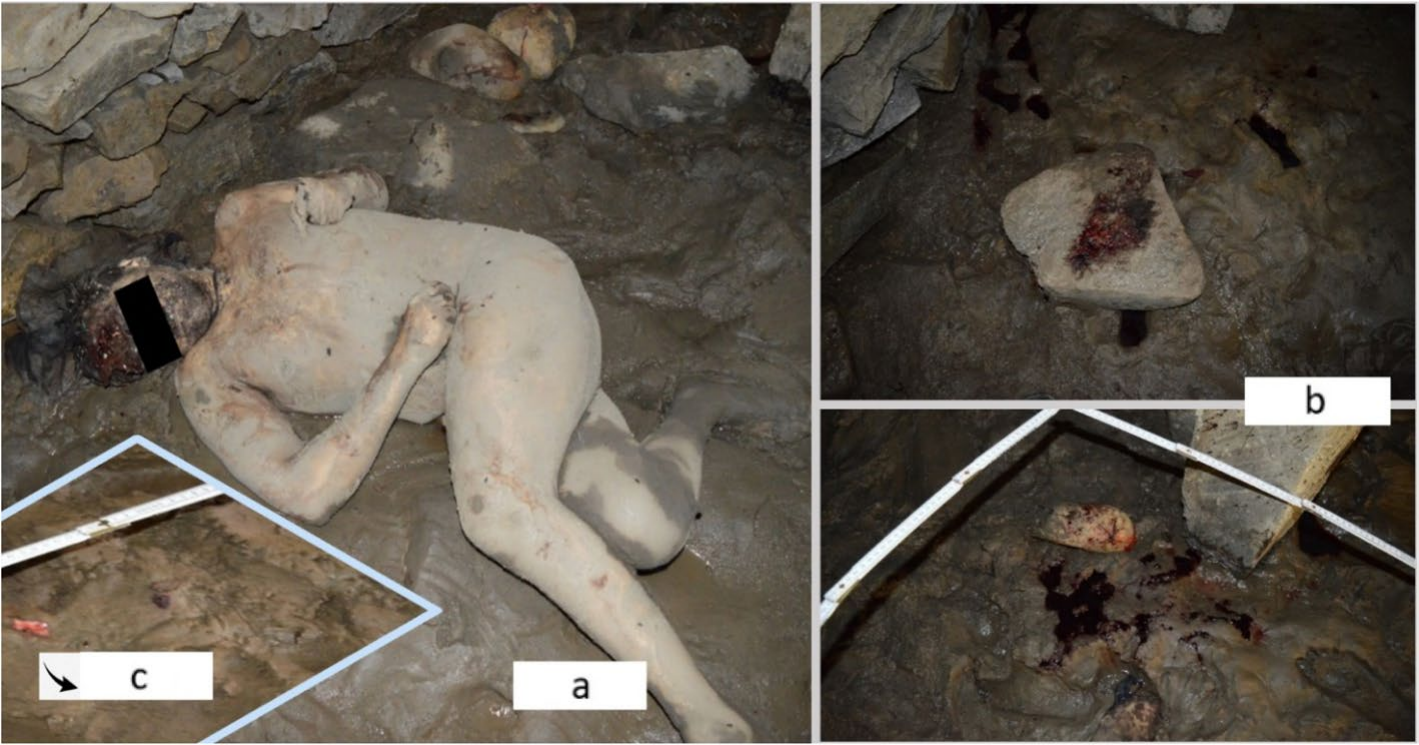
Details from the on-site investigation: he was found naked in the basement (**a**); muddy ground with protruding stones - some of them blood-stained (**b**); a small fragment of the frontal bone (**c**)

A 26-year-old man from a rural area was found dead in 2021 (the second year of the COVID-19 pandemic) at his parents’ house (where he usually lived alone), barricaded in the basement (Fig. 1a). He was naked, covered with a thin layer of dry mud, with his feet in the damp earth. The ground was covered with mud and protruding stones, and some of the stones were blood-stained (Fig. 1b). He had craniofacial trauma with multiple lacerations of the scalp and frontal fracture with a lack of frontal bone substance, torso trauma, and other injuries (bruises and abrasions) on the limbs. Initially, due to the presence of these multiple lesions (some of them atypical for a committed suicide), a homicide was suspected. The on-site investigation revealed a small fragment of the frontal bone lying on the ground (Fig. 1c). The preliminary psychological autopsy highlighted a psychopathological profile, with a history of schizophrenia. He did not leave a farewell note. His mother visited him 2 days before the corpse was found. Between the time of her visit and the moment the corpse was found, no one saw him or could get in contact with him. His mother thought he did not want to communicate with the family, so she returned after 2 days and called the police.

## Materials and methods

Following the on-site investigation, a forensic autopsy was performed. This included histopathological and toxicological investigations such as the level of blood-alcohol, urine-alcohol, and general toxicologic examination of blood, using gas chromatography-mass spectrometry (GC–MS). Additionally, we applied the psychological autopsy method. We collected relevant information from his mother using a specially designed questionnaire based on two types of questions. The first ones were open-ended questions designed to facilitate an open discussion about the events that marked the life of the deceased, the interpersonal relationships (both with the family and with other people), questions related to alcohol consumption, or compliance with psychiatric treatment. We also used specific questions meant to identify the potential role played by the COVID-19 pandemic in the deterioration of his mental state. The second type of questions consisted of closed-ended ones, gathering epidemiological and medical history data (psychiatric records, suicidal ideation, and possible suicide attempts). At the same time, we tried to identify the behavioral changes of the deceased, both in long-term and recent antecedents: (1) communication of suicidal ideation, (2) sadness, (3) the tendency to isolation, (4) anxiety, (5) aggressiveness, (6) nervousness, (7) insomnia, (8) chronic fatigue, (9) lack of interest to participate in family life, (10) lack of interest to integrate into social life, (11) uselessness, (12) culpability, (13) self-depreciation, (14) insecurity, and (15) loss of the interest to live. The confidentiality and ethical considerations were respected, following the legislation on data protection. The abovementioned data was collected to analyze the behavioral changes, motivations, and risk factors of this schizophrenic person, that finally lead to committed suicide.

The specific findings of our case were corroborated with risk factors and behavioral changes described in references within literature databases, using the keywords “committed suicide,” “suicide attempt,” “schizophrenia,” “COVID-19 pandemic,” and “atypical suicide.”

## Results

### Findings at the forensic autopsy—external examination

The corpse had livor mortis (imbibition stage), cadaveric rigidity that could be defeated more easily, and the corpse was without any sign of putrefaction.

**Fig. 2:**
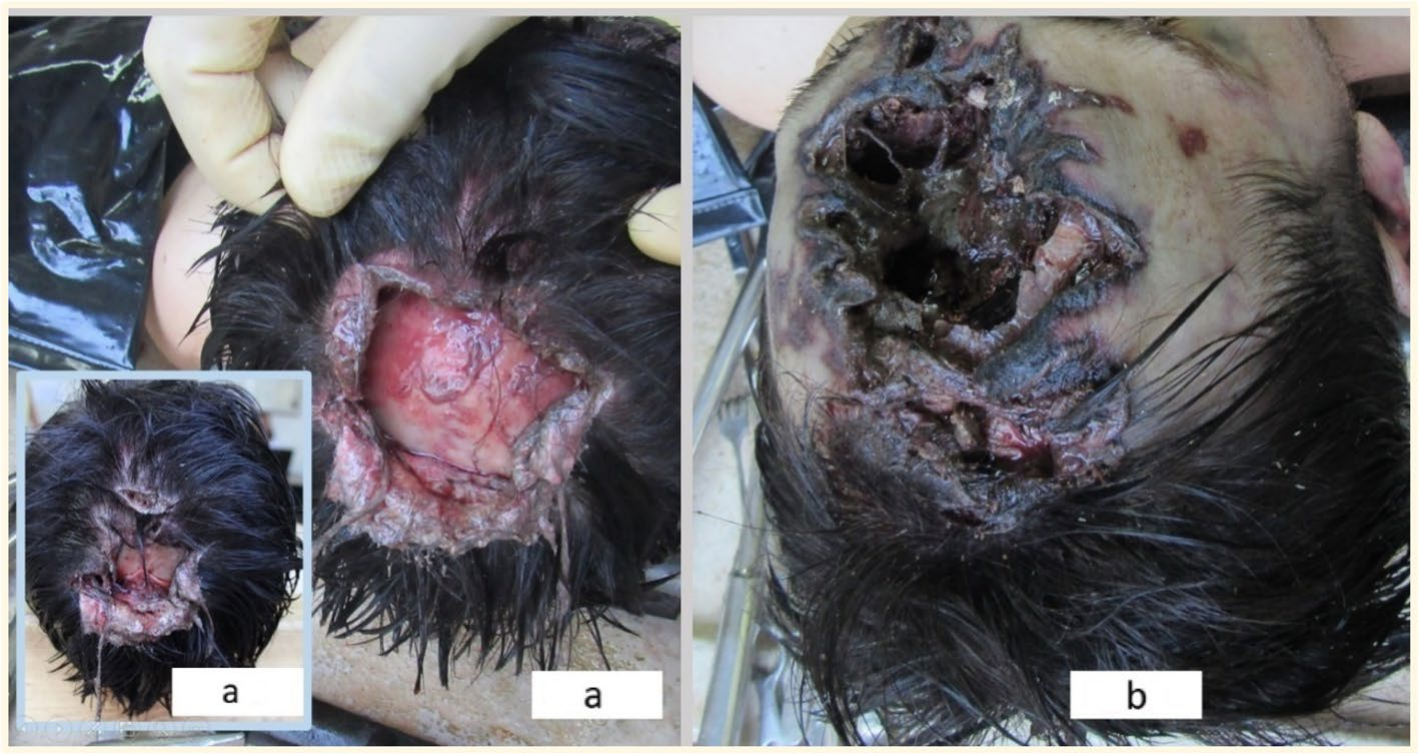
The complex craniofacial trauma - multiple lacerations of the scalp (**a**, **b**); frontal laceration with underlying fracture (**b**)
- (2) Four superficial stab wounds of the torso (Fig. 3a) spread over an area of 10/6 cm: three of them at the anterior base of the right hemithorax (up to 1.3-cm long, with regularly cut black-brownish edges), and a fourth situated in the right hypochondrium (0.8-cm long, with the same characteristics).

- (1) Multiple lacerations of the scalp, with torn edges, with various locations (Fig. 2): (1a) vertex—4/3 cm, with a posterior skin flap, and no underlying fractures (Fig. 2a); (1b) parietal—midline, 3/0.4 cm, no underlying fractures (Fig. 2a); (1c) frontal—5-cm long, situated at the limit of hair insertion, with edges that appeared torn, reddish-gray, with marginal parchment-like blackish aspect (Fig. 2b); and (1d) at the center of the frontal region (9/7 cm, lacking skin substance, with multiple skin flaps at the periphery, and with parchment-like black-brown edges). The latter was part of a complex trauma (Fig. 2b) that included a fracture of the underlying frontal bone, with a missing fragment of 8/6 cm that allowed the visualization of local intact gray-blackish dura mater.

**Fig. 3:**
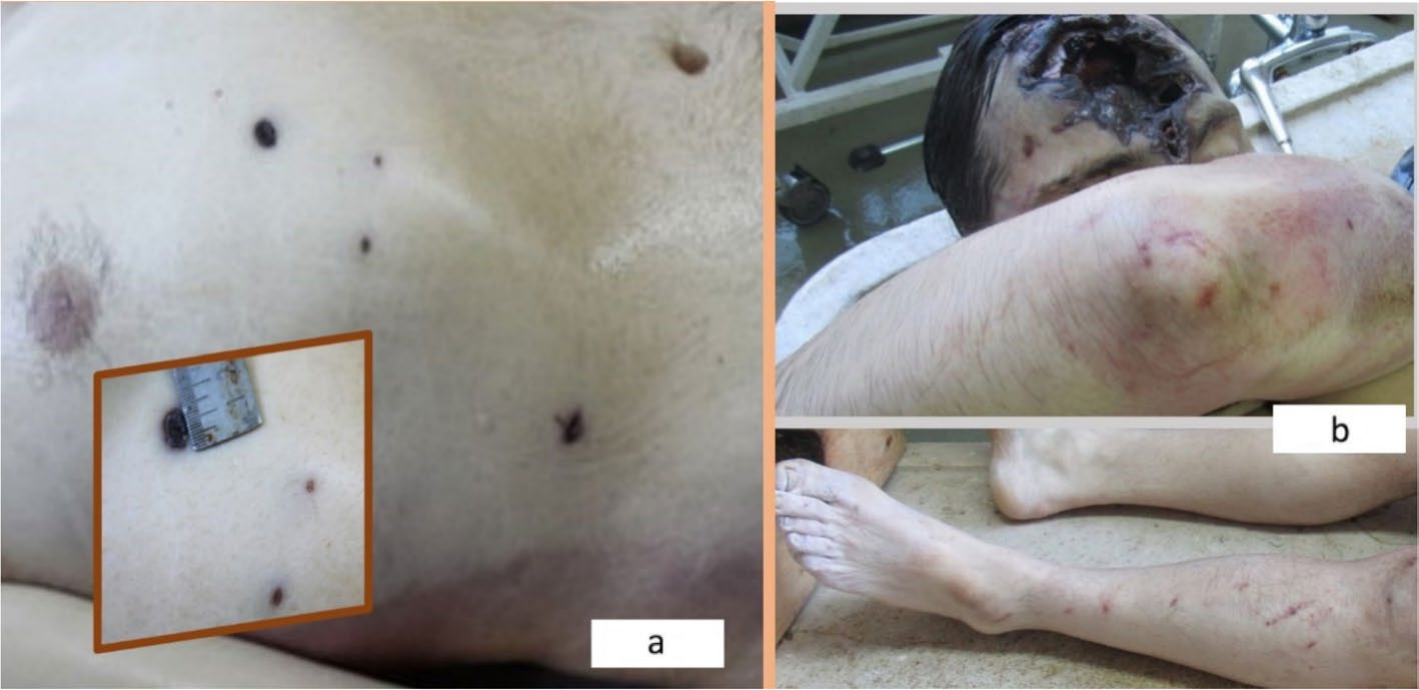
The superficial stab wounds of the torso (**a**); bruises and abrasions of the limbs (**b**)
- (3) Multiple bruises and abrasions on the limbs (Fig. 3b). Hyper hydration of the soles was also present (his feet were covered with wet mud).

### Findings at the forensic autopsy—internal examination

- (1) Head: dark red epicranial hemorrhagic infiltration (corresponding to all the wounds described at the external examination), the frontal fracture described above at the external examination, subarachnoid hemorrhage (predominantly frontal), and acute cerebral edema and frontal cortico-subcortical cerebral contusions (numerous red-blackish spots with wedge-shape arrangement with the widest part in the outermost part of the brain, occupying an area of about 3 cm in diameter), corresponding to the complex frontal trauma. No epidural or subdural collections were found. The dura mater was intact, presenting a gray-blackish area of about 9 cm in diameter, at the center of the frontal part.
- (2) Thorax and abdomen: minimal hemorrhagic infiltration (up to 2 cm in diameter) underlying the wounds described at the external examination; acute pulmonary emphysema (pulmonary pale areas of air space enlargement on the surface, that appear drier at the section); acute pulmonary edema (dark red blood mixed with pink air foam); hypertrophic cardiomyopathy (dimensions of 15/12/6.5 cm, with a left ventricular wall thickness for up to 2.4 cm); a few Visnevski spots (brown-blackish, scattered especially along the small curvature of the stomach, up to 0.3 cm in diameter); and steatotic hepatic dystrophy (slightly enlarged liver with a rounded anterior edge, red-brownish with yellow areas).

### Additional laboratory examinations

The histopathological findings confirmed the macroscopic aspects revealed above. We found a 0.13‰ grams blood alcohol concentration, and urine-alcohol was negative. The general toxicologic examination performed was negative (including substances used in the treatment of schizophrenia).

### The psychological autopsy findings

These data were collected at the crime scene but also by using the specially designed questionnaire.

#### Socio-economic and general data

We found out that the victim was a young man (26 years old), of Romanian nationality and Christian Orthodox religion, living in a rural area, celibatarian, with secondary education, recipient of a disability pension for schizophrenia, with a minimal income. He lived most of the time alone in his parents’ house and was an introverted person.

#### Psychiatric history

He was diagnosed with schizophrenia almost 10 years ago, with multiple psychiatric hospitalizations. He was under outpatient treatment; recent low therapeutic compliance was described, with incomplete acceptance of recommended treatment. He had two prior suicide attempts, one by self-inflicted wounds and another by trying to “take his eyes out”. Otherwise, he was not known for other medical conditions.

#### Drinking habits

He used to drink alcohol daily, most often beer and wine.

#### Motives

The possible reasons with a potential role in triggering the suicidal act were described as follows: (1) illness and disability (awareness of the disease) and (2) family quarrels.

#### Behavioral changes (old and recent signs)

From the distant background of this schizophrenic patient, the mother selected the following signs: communication of suicidal ideation, anxiety, nervousness, aggressiveness, lack of participation in family life, lack of social life, uselessness, culpability, and self-depreciation. During the COVID-19 pandemic, he became very religious and went to church frequently. Right before his death, he exhibited the following signs: he communicated suicide ideas more frequently, he became more anxious, and he wanted to be isolated from everyone. His mother noticed that lately, he refused the medication prescribed by the psychiatrist.

**Fig. 4:**
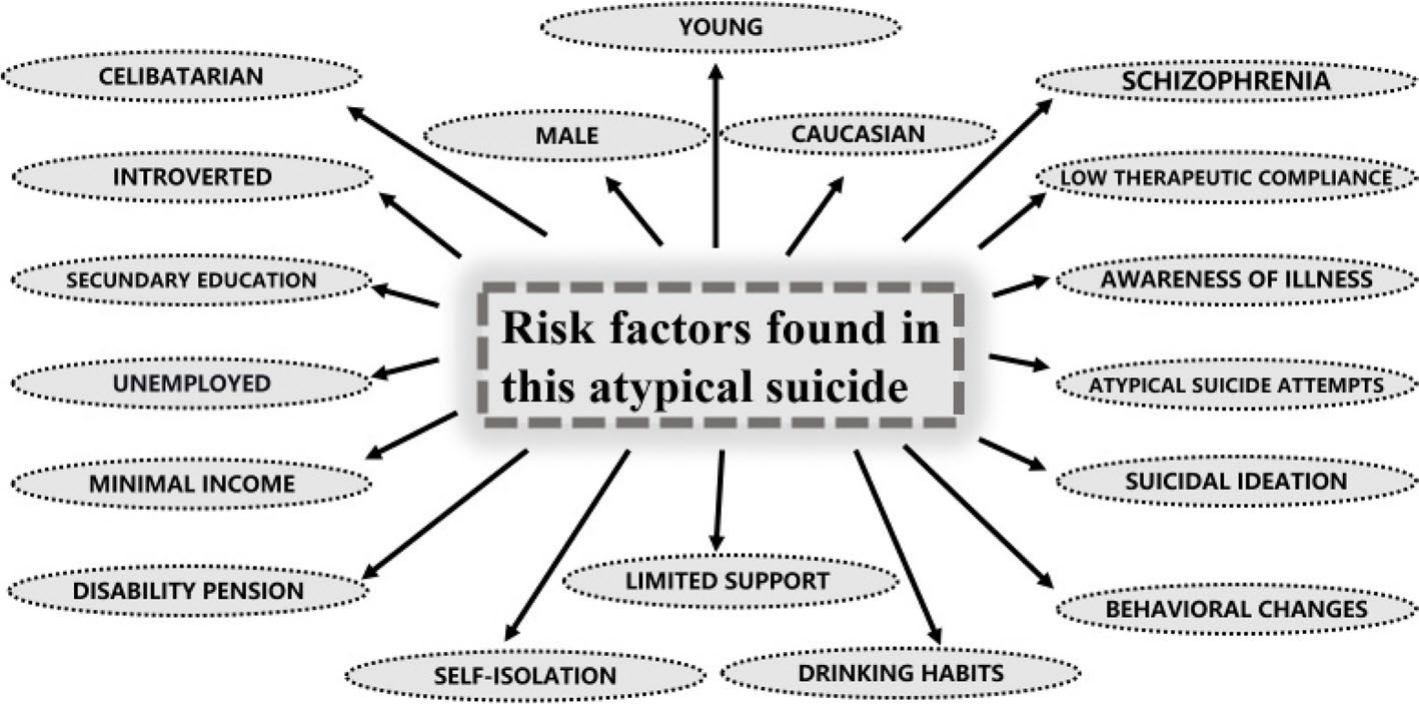
Risk factors for suicide found in this particular case

Based on these findings, we identified the following risk factors for this particular case (Fig. 4):

*Following the complete forensic autopsy and by using the data provided by the psychological autopsy*, we concluded that death was violent in nature and was caused by a severe acute craniocerebral trauma, with external and meningo-cerebral hemorrhage. We decided based on our findings that the death occurred 2 days before he was found dead. The traumatic head injuries were self-inflicted and were the result of repeatedly hitting his head against blunt objects with irregular surfaces; these injuries had a decisive role in the occurrence of death. The traumatic injuries of the torso were also self-produced (by prior self-stabbing); these injuries did not play a role in the occurrence of death. The lesions of the limbs were most likely the result of accidental hitting against blunt objects and also did not play a role in the occurrence of death.

## Discussion

### Forensic considerations

The contribution of forensic medicine in investigating committed suicide is undeniable, as it establishes the manner of death and the suicidal method. According to Romanian legislation, all suicide cases are subject to a forensic autopsy that allows suicides to be differentiated from other forms of violent death (by a rigorous differential diagnosis with hetero-aggressions and accidents), but also from nonviolent (pathological) deaths.

The peculiarity of this case results not only from the atypical character of the suicidal method but also from the fact that this method was doubled (preceded) by another relatively rare suicidal method (self-stabbing). This method (used in a previous suicide attempt) proved to be ineffective this time as well, requiring the use of an alternative method (more aggressive and more atypical).

Thus, our subject repeatedly hit his head against irregularly shaped blunt objects, resulting in a severe self-produced craniocerebral trauma. Atypical suicides are described in the literature in schizophrenic patients, such as the case of a male adult with schizophrenia who committed suicide by inserting a teaspoon into the respiratory tract (Turla et al. 2007).

In this case, the differential diagnosis with hetero-aggression was very important, given that the lesions found at the autopsy were numerous, spread in various anatomical regions, and were produced with different vulnerable agents—aspects commonly encountered in aggressions. Additional forensic laboratory examinations were very useful. Microscopic examination proved the vital character of the injuries (proving their antemortem occurrence).

The negative general toxicologic examination (including for substances usually used in the treatment of schizophrenia) indicated that he had not received treatment for schizophrenia. Lack of treatment could have led to mental degradation that increased the suicide risk. Toxicology revealed only a 0.13‰ g of blood alcohol level, making an accidental fall after alcohol ingestion very unlikely. Generally, postmortem synthesis of alcohol is usually less than 0.07 g/100 ml, but it depends on several factors (postmortem interval, temperature, environmental conditions, body trauma); however, higher concentrations (greater than 0.1 g/100 ml) have been reported in data literature (Honey et al. 2005). Thus, in our case, because the putrefaction has not occurred and the corpse was found in the basement at a low temperature (Athanaselis et al. 2005), we have considered that the value of alcohol detected at autopsy is due to acute alcohol consumption (urine-alcohol examination was negative).

The data obtained during the on-site investigation and those provided by the forensic autopsy may (and should) be corroborated with the information provided by the psychological autopsy. Based on these findings, a positive diagnosis of suicide can be established. In our case of atypical suicide in a person known to have schizophrenia, the psychological autopsy was very useful in investigating the psychiatric past, highlighting the psychopathological tension of the subject, outlining and understanding the motives and the chosen suicide method, and evaluating the distant and recent behavioral patterns. Thus, a psychological autopsy can be useful for reaching a positive diagnosis of suicide in people who were diagnosed with schizophrenia (Hădărean et al. 2011). The investigation of the psychiatric past (by applying a specially designed questionnaire) is an important element for shaping the positive diagnosis of suicide, completing the objective forensic criteria.

### Susceptibility of schizophrenics for committing suicide

In schizophrenic patients, the risk of suicide is higher (Hettige et al. 2018; Kasckow et al. 2011), suicide deaths being common (Tousignant et al. 2011). Thus, suicide is an important cause of death among patients with schizophrenia (Gómez-Durán et al. 2012; Togay and Lippmann 2019). Literature data indicate that at least 5–13% of schizophrenic patients die by suicide, and these figures may be even higher (Pompili et al. 2007).

Literature also provides data that suggests that in people with schizophrenia the risk of suicide is high throughout their life, and usually suicide occurs at the beginning of the disease. Young patients who are in the early stages of being diagnosed with schizophrenia are more likely to commit suicide than older patients with schizophrenia, so prevention and intervention efforts should be directed to the early stages of the disease (Palmer et al. 2005). There are also studies that suggest that suicides can be seen at any stage of the illness in patients with schizophrenia (Sinclair et al. 2004). In our case, the patient was at the end of the first decade of schizophrenia. Suicide in the first decade is suggested to be more frequent (Togay and Lippmann 2019; Tsuang and Woolson 1978); for example, in a study of 200 patients with schizophrenia that were monitored, 44% committed suicide in the first decade of illness (Sher and Kahn 2019).

### Psychological autopsy findings

#### Socio-economic risk factors in schizophrenia

Predicting suicide in patients with schizophrenia is difficult and complex (Hor and Taylor 2010). Suicide risk predictors involve many factors (Brådvik 2018). In our case, by using the psychological autopsy, we found the following risk factors for suicide: young Caucasian male, celibatarian (Balhara and Verma 2012), unemployed, aware of his disease, with suicidal ideation, and a history of suicide attempts, self-isolated, with minimal external or family support (with some family tensions and conflicts), with feelings of uselessness, with low compliance to treatment, and daily alcohol consumption. Identifying risk factors can be helpful for the prediction and prevention of suicide. Our case revealed many of the demographic and psychosocial risk factors mentioned in the literature (Sher and Kahn 2019; Chan et al. 2016; Pompili et al. 2007; Carlborg et al. 2010; Siris 2001; Isometsä 2014).

#### Self-harm, suicide attempts, and suicidal motivation

The suicidal method in patients with mental disorders is often unusual, as it was in the presented case where extreme self-harm resulted in committed suicide. Patients with mental disorders may have a history of suicide attempts or self-harm. Data obtained by applying the questionnaire revealed prior aggressive and atypical self-harm (“he tried to take his eyes out”) and also his preference for self-inflicted stab wounds. Self-harm is defined as the action through which a person injures his/herself in an impulsive, intentional, and repeated manner. Self-harm does not aim to end one’s own life, even though it can coexist with suicidal ideation. We noticed that among the most common self-injury methods are the same ones used in this specific case, namely hitting the head (21–44%) and cutting the skin (70–90%) (Pompili et al. 2015). Some studies on self-harm prevention efforts in patients with schizophrenia support careful monitoring of mental state, adherence to treatment, and avoidance of alcohol consumption (Chan et al. 2016), which in our case were all ignored.

There is data in the literature that confirms the important role of the psychological autopsy method in emphasizing the reasons for committing suicide (Majid et al. 2017). Following the application of the questionnaire, we were able to detect as suspected motives of suicide the fact that he acknowledged his disease and that he was involved in family conflicts. These issues seem to have been able to unbalance his mental state. A cross-sectional study of 388 patients with schizophrenia (Mork et al. 2012) found that self-harm in men was associated with their awareness of having a psychiatric disorder. Awareness of psychiatric illness has been associated with self-harm only in men (Chan et al. 2016), which applies to our case as well. Suicide attempts are also considered important risk factors for suicide (Sher and Kahn 2019).

#### Alcohol consumption in schizophrenia increases the potential risk for suicide

Alcohol consumption plays an important role in facilitating the recourse to the act of suicide by reversing cortical inhibition, increasing impulsivity, and encouraging self-harm (Pompili et al. 2010; Kendall 1983; Sher 2006). Research conducted to determine the level of alcohol in the blood of those who committed suicide shows the presence of alcohol at a rate of 10–54% (Bilban and Skibin 2005). The risk of suicide is much higher in people with known schizophrenia and alcoholism (Mann 2002). We were able to document in our case a blood alcohol level of 0.13‰ grams, as well as the association between regular alcohol consumption, schizophrenia, and committed suicide.

Alcohol exacerbates the symptoms of depression, anxiety, or bipolar disorder by lowering the level of dopamine, which can trigger an increase in self-harm (Design for recovery, substance abuse and self-harm 2019) which is a risk factor for suicide (Hawton and Heeringen 2009).

#### Behavioral changes (before and during the COVID-19 pandemic, and right before committing suicide)

##### Warning signs identified before the COVID-19 pandemic

According to his mother, he considered himself useless, guilty, and inferior. He had moments of anxiety and episodes of nervousness and aggression. As literature shows, patients with schizophrenia, who committed suicide, are more likely to have had more aggressive behavior (Pompili et al. 2010). Once in a while, he communicated the ideas of suicide and he has been showing low interest in family and social life participation. Despite these signs, he received proper treatment and was well monitored by health professionals. In this period, it is possible that suicide did not occur because he was monitored psychiatrically at the beginning and during the course of the illness (multiple hospitalizations with treatment compliance), although suicide ideation was present. There is a lower risk of suicidal behavior during carefully monitored treatment of psychiatric patients, so treatment adherence is an important factor in preventing suicide (Chan et al. 2016).

##### Behavioral changes identified during the COVID-19 pandemic

Our remarks on the subject’s behavioral changes point out that his spiritual beliefs were enhanced (he became very religious and went frequently to church). He did not follow the treatment correctly, and the symptoms worsened progressively. During the COVID-19 pandemic, access to the system health and mental health services was restricted (Micluţia 2021) and the vulnerability to stressors is greater (Esposito et al. 2021). We believe that during the COVID-19 pandemic, special attention must be paid to psychiatric patients, especially if they have manifested prior suicidal behavior. A study on psychiatric services in Romania showed that during the COVID-19 pandemic, new types of delusions and behaviors were noticed, and several patients with schizophrenia adapted their delusions to this ongoing pandemic (bizarre ideas of contamination, divine messages about rescuing humanity combined with other mystical delusions) (Micluţia 2021). Other studies have shown that the immediate effects of the COVID-19 pandemic on schizophrenic patients are persecutory delusions and visual hallucinations (Seifert et al. 2021). Delusion in schizophrenia is episodic (Arantes-Gonçalves et al. 2018). The danger of suicide in schizophrenics is heightened by delusional ideas and especially by imperative hallucinations that could lead to suicide (Hădărean et al. 2011). Negative consequences are to be expected in this pandemic situation for the mental health of those with known schizophrenia (Kozloff et al. 2020 Jul 8; Hamada and Fan 2020; Panariello et al. 2021; Mohan et al. 2021). Also, some studies affirm the role of the COVID-19 pandemic in the development of psychotic symptoms in schizophrenic patients (Barlati et al. 2021; Brown et al. 2020). In our case, possible because he lived alone, we could not document persecutory delusions and visual hallucinations, although all of the above suggest some behavioral changes, further studies are needed to confirm the hypothesis that the COVID-19 pandemic could trigger intense psycho-emotional stress or could decrease the mental stability of people with schizophrenia.

##### Warning signs identified before committing suicide

Some of the signs were intensified. He expressed more often suicidal ideation, he became more anxious, and he wanted to be totally isolated from everyone. Literature data highlights increased suicidal risk in patients with schizophrenia who have expressed suicidal ideation (Harmer et al. 2021). He stopped consulting a psychiatrist, and therefore, the treatment was not administered properly. The behavioral changes we identified for our specific case (lack of therapeutic compliance, poor socioeconomic status, minimal family support, and other risk factors highlighted above) led to a significant increase in suicide risk and determined a significant deterioration of his mental status, as other studies suggest (Harmer et al. 2021; Turla et al. 2007). The presented case (suicide performed by using an atypical method) shows the variability over time of the psychopathological picture in patients with schizophrenia, especially if a lack of therapeutic compliance is identified. In committing suicide, the most important protective factor in schizophrenic patients is treatment adherence (Sher and Kahn 2019). Moreover, these patients might benefit from neurofeedback procedures to alleviate their condition, in addition to psychiatric treatment (Hammond 2005; Pérez-Elvira et al. 2021a, b; Lee et al. 2019).

## Conclusions

The case we chose to present stands out by the atypical suicidal method that was chosen, in direct correlation with the underlying psychiatric pathology of the subject. Even though there were elements that pointed out a possible homicide, the on-site findings, the forensic autopsy, and the psychological autopsy gathered sufficient information to outline the positive diagnosis of suicide, allowing a rigorous differential diagnosis with hetero-aggression and accidental injury. We emphasize the important role of the psychological autopsy, which pin-pointed the motives and the risk factors associated with this particular suicide case in a schizophrenic patient, in accordance with literature data. We consider that this method should be regularly used in the practice of suicide investigation since it allows not only the drawing up of a suicide-positive diagnosis but also can provide a detailed picture of mental degradation and the associated suicide risk factors. The specially designed questionnaire that we used can be very useful in this regard. Last but not least, the presented case is suggestive of the importance of specialized medical treatment in psychiatric patients, especially regarding schizophrenia—a mental disease with unpredictable evolution and with the possible suicidal outcome. In our opinion, this case also suggests the possible role of the COVID-19 pandemic in triggering committed suicide in schizophrenic patients as a result of limited access to psychiatric facilities and treatment.

## Supplementary Information

**Additional file 1.**

## Data Availability

All data and materials used during this study are included in this article.

## Abbreviations

GC-MS: Gas chromatography-mass spectrometry
COVID-19: Coronavirus disease 2019
