# Supplementary material for: The role of psychological autopsy in investigating a case of atypical suicide in schizophrenia: a case report with a brief review of literature
Source: Egypt J Forensic Sci. 2022 Jul 6;12(1):30. doi: 10.1186/s41935-022-00291-5 (PMC9257119; doi:10.1186/s41935-022-00291-5)
Supplement: Supplementary file 1 — Additional file 1. [file 41935_2022_291_MOESM1_ESM.docx]

**By completing the questionnaire, you agree to provide the data necessary for the study.**

The data obtained by completing the questionnaire will be used strictly in research on suicide. Confidentiality of data is assured. The study does not imply any risk to your physical and/or mental integrity. If you have any questions, please contact the person in charge of the project, Roxana-Mihaela Crișan (contact email: crisanrox@gmail.com), who Ph.D. student at ULBS.

No. AUTOPSY …………….............. AUTOPSY DATA ..................................... SIBIU / MEDIAŞ

GENDER………AGE……

RESIDENCE……………………………………………………..URBAN/RURAL .........

OPEN DISCUSSION -major events

- interpersonal relationships

- accohol consumption/ compliace with psychiatric treatment

- COVID-19 pandemic - changes?

I. CIVIL STATUS:

1. married (first marriage)

2. second marriage

3. third marriage

4. singer/celibatarian

5. widower

6. divorced

7. concubinage

II. NUMBER OF CHILDREN …...of which: minors ..........adults ……….married………employed…

III. OCCUPATION: 1. preschooler, 5. unemployed (how many years?……….),

2. schooler, 6. retired (how many years?………..),

3. student, 7. employee (what worked?…………….).

4. housewife,

IV. PROFESSION……………….........................….STUDIES……………………………………………

NATIONALITY…………………………………….RELIGION…………………...............……………..

V. DECEASED'S INCOME (how much did he earn per month?)…… …..

The total income of the persons with whom he lived……………….

VI. DATE OF DEATH: day…..., month……, year……, hour.......

VII. PLACE OF DEATH:

1. at home

2. at work

3. in hospital

4. psychiatric hospital

5. în the army

7. elsewhere…………

VIII. INCURABLE DISEASES FROM WHICH HE SUFFERED……………………………...

NEURO-PSYCH DISEASE: 1. did not suffer 5. Depression/maniac-depressive psychosis

2. alcoholism 6. Psychopathy/personality disorders

3. epilepsy 7. Other mental illnesses

4. schizophrenia 8. Cannot specify the type of illness

IX. INPATIENTS FOR PSYCHIC DISEASES: …................…………………………………………….

X. OTHER SUICIDE ATTEMPTS? (if so, how many? In what way?)…………………………

XI. CASES OF SUICIDE IN THE FAMILY OF THE DECEASED?

1. no

2. to parents

3. to grandparents

4. to brothers

5. primary cousins

6. to other relatives( what kind?)……

XII. GOODBYE LETTER YES....... NO.........

XIII. DID HE CONSUMES ALCOHOL?

1. daily,

2. 2-3 or a week,

3. once a week,

4. occasional,

5. not at all.

XIV. WHAT KIND OF ALCOHOL DID HE CONSUME MOST FREQUENTLY?………...............……….

DID HE CONSUME ALCOHOL ON THE DAY OF HIS DEATH?……YES………NO ………what?….....

BLOOD-ALCOHOL AT AUTOPSY …………………………………………………………………….

XV. WHAT MOTIVES DO YOU THINK HE HAD FOR COMMITTING SUICIDE?

1. has been intimidated/bullied/terrorized in any way (at school, at work, etc.)

2. school problems

3. emotional problems (quarrels with boyfriend/girlfriend)

4. family conflicts (quarrels, jealousy, etc.) …………………………………….

5. conflicts with other people (what kind?) …………………………………………

6. material problems (debts) …………………………………………………

7. death or illness of a close person ………………………………….

8. own illness or infirmity ……………………………………………….

9. job loss (unemployment, dismissal, etc.) ……………………….

10. professional failures (problems at work)

11. judicial problems (under investigation by the police, sentenced to prison, payment of fines, etc.)

12. problems of sexual life

13. COVID-19 pandemic?

13. other reasons ...........................................................................................

XVI. How do you think the COVID-19 PANDEMIC influenced the imbalance of the deceased's mental state?

1. SARS-CoV-2 infection with hospitalization
2. SARS- CoV-2 infection with isolation
3. Loss of service
4. Lack of money
5. Loss of a family member
6. Fear of illness/hospitalization
7. Lack of social activities
8. It was not influenced

XVII. WHICH OF THE FOLLOWING SIGNS DID THE DECEASED EXHIBIT?

1. communication of suicidal ideation

2. sadness

3. the tendency to isolation

4. anxiety

5. aggressiveness

6. nervousness

7. insomnia

8. chronic fatigue

9. lack of participation in family life

10. lack of interest to integrate into social life

11. uselessness

12. culpability

13. seff-depreciation

14. insecurity

15. loss of the interest to life

XVIII. WHAT CHANGES IN BEHAVIOUR DID HE HAVE IN THE PERIOD RIGHT BEFORE COMMITTING SUICIDE (choose from the 15 points above) …………………………………….

Possible pandemic-induced changes ...................................................................

XIX. Who completed the questionnaire?

1. wife/husband 4. other relatives

2. children 5. neighbors/ acquaintances

3. parents 6. other persons ........

XX. OTHER COMMENTS (other important data that were not mentioned above) ..................................

…………………………………….......................................………………………………………………………………….......................................................................…………………................................……
